# Identifying Evacuation Needs and Resources Based on Volunteered Geographic Information: A Case of the Rainstorm in July 2021, Zhengzhou, China

**DOI:** 10.3390/ijerph192316051

**Published:** 2022-11-30

**Authors:** Jingyi Gao, Osamu Murao, Xuanda Pei, Yitong Dong

**Affiliations:** 1 Department of Architecture and Building Science, Graduate School of Engineering, Tohoku University, Sendai 980-8579, Japan; 2 International Research Institute of Disaster Science, Tohoku University, Sendai 980-8572, Japan; 3 Department of Earth Science, Graduate School of Science, Tohoku University, Sendai 980-8578, Japan

**Keywords:** disaster prevention, volunteered geographic information, evacuation needs, rainstorm, latent dirichlet allocation model, random forest

## Abstract

Recently, global climate change has led to a high incidence of extreme weather and natural disasters. How to reduce its impact has become an important topic. However, the studies that both consider the disaster’s real-time geographic information and environmental factors in severe rainstorms are still not enough. Volunteered geographic information (VGI) data that was generated during disasters offered possibilities for improving the emergency management abilities of decision-makers and the disaster self-rescue abilities of citizens. Through the case study of the extreme rainstorm disaster in Zhengzhou, China, in July 2021, this paper used machine learning to study VGI issued by residents. The vulnerable people and their demands were identified based on the SOS messages. The importance of various indicators was analyzed by combining open data from socio-economic and built-up environment elements. Potential safe areas with shelter resources in five administrative districts in the disaster-prone central area of Zhengzhou were identified based on these data. This study found that VGI can be a reliable data source for future disaster research. The characteristics of rainstorm hazards were concluded from the perspective of affected people and environmental indicators. The policy recommendations for disaster prevention in the context of public participation were also proposed.

## 1. Introduction

### 1.1. Background

Climate change has led to extreme rainfall and the resulting floods have been frequent in recent years, severely hindering global sustainability [1]. Populations and economic activities are concentrated in cities and hence vulnerable to heavy rainfall and urban flooding [2]. In many developing countries [3], urbanization has considerably impacted hydrology [4]. For example, the extreme rainstorm event in Zhengzhou, China, in July 2021 resulted in a large number of casualties and property damage. The mismatch between rapid urbanization and infrastructure construction, such as underdeveloped urban road networks [5], has increased the likelihood of urban flooding from heavy rainfall [6]. In recent years, urban flooding caused by extreme rainstorms has frequently occurred in many countries; since urban rainstorms are sudden, clustered, and continuous, emergency management is extremely difficult [7]. How to reduce the impact of extreme weather, especially urban rainstorm hazards has become an important topic in the field of Disaster Risk Reduction (DRR). The Sendai Framework for Disaster Risk Reduction 2015–2030 (SFDRR) emphasized the priority of understanding disaster risk. To achieve this, it is necessary but not limited to collecting, and analyzing the relevant data. Besides, the use of location-based disaster risk information and related technology is encouraged to realize the identification of disaster vulnerability, characteristics, etc [8].

Technological development and the Web 2.0 era provide unlimited possibilities for DRR and emergency management (EM). Emergency management is a complex field that involved multi-disciplinary, interdisciplinary approaches, and a diversity of data and information. Big data and emergency management (BDEM), is now viewed by researchers as an emerging research area based on different knowledge, culture, and social backgrounds. Applications such as mobile communication, online social network, etc. are the core topics of the BDEM field [9]. Information visualization (InfoVis) is another key issue in EM. It provides an approach to helping to understand and analyze the huge amount of data produced during emergencies [10]. Based on the visualization of the multi-source information, the government will be easier to make the appropriate decisions in a disaster. The emergence of volunteered geographic information (VGI) and its widespread use have greatly helped disaster research and EM.

VGI refers to geographic information that is voluntarily and widely created and shared by citizens through platforms such as social media and mobile smart apps [11]. Created primarily by common citizens, these data have recently emerged as a complementary information source to traditional authoritative information [12]. Network technology development and the use of devices such as smartphones have enabled citizens from all literacy and age backgrounds to play the role of sensors, receiving and generating various spatial information from their daily lives. This spontaneously generated VGI is widely used in environmental monitoring, event reporting, disaster management, human behavior analysis, and land-use mapping [13,14]. VGI has also been widely used to understand and analyze the development of cities or regions, as well as human activities [15]. It is as effective as professional geographic information (PGI) in an outdoor recreational context, and the combination of PGI and VGI can be effectively used for the planning of outdoor activities [16]. In disaster-related research, VGI plays a critical role in the disaster management processes of prevention, preparedness, and response. Most studies have focused on the application of citizen-contributed VGI data in disaster response [17,18], and it has been proven to be very useful for collecting disaster crisis information, especially in flood and fire disasters [19]. Highly credible on-site location information is critical for rescue and subsequent recovery during disasters; however, such data are often difficult or impossible to obtain in a timely manner. VGI is highly valuable for the affected population, including for rescuers, decision-makers, and other key players [20]. The generation, dissemination, and use of VGI for disaster-related geographic data provided by citizens are realized through the development of VGI-related technologies and volunteer assistance [21,22]. VGI is cost-efficient and of lower cost than methods of traditional data collection and use [23]. It can provide near real-time information during disasters and location information that can be used to effectively identify disaster risk areas and generate hazard maps [24]. In addition to disaster response, it can also be applied to post-disaster spatial planning. For example, Kusumo, Reckien, and Verplanke [25] used VGI to analyze the choice and preference of shelter locations during floods among residents of Jakarta, Indonesia and compared it to the locations of official evacuation shelters. VGI is emerging as a new data source that can be applied to urban resilience enhancement, which breaks the traditional top-down resilience enhancement pathway [26] and enhances participation during disaster management in a bottom-up manner, contributing toward the resilience of affected groups and regions [27,28].

Research and application of VGI are largely technology-driven [29]. The application and development of VGI data are largely constrained by their inherent uncertainty and the need for extensive manual manipulation. Therefore, effective VGI use requires support from other technologies [30]. The combination of artificial intelligence (AI) related methods in VGI data application has become an important research trend in recent years. For example, Yuan et al. [31], used VGI data and deep-learning methods to map buildings in Kano state, Nigeria, to effectively support socio-economic development. Arapostathis [32], used machine learning to classify information from VGI-sourced social media tweets to identify information (e.g., geographic location) for flood disaster research. Feng and Sester [33], combined deep learning to analyze flood events in Paris, London, and Berlin. It is difficult for managers and policymakers such as governments to collect statistical data on natural disasters such as typhoons. However, real-time disaster information shared by citizens through social media platforms during a disaster can generate a considerable amount of VGI regarding the disaster situation. This provides effective information for disaster management stakeholders. Its proper use can also strongly support disaster risk reduction [34]. For example, VGI data from social media and the K-nearest neighbor (KNN) algorithm were used to extract and classify typhoon disasters in the southeastern coastal region of China [35]. Currently, VGI is predominantly applied to flood and forest fire studies in Europe and North America; the combination of VGI data and scientific models has become an important research method for natural hazard analysis in recent years [36].

### 1.2. Recent Trends for Rainstorm Research

The research methodology for determining flooding risk has recently shifted from qualitative to quantitative studies [37]; however, most studies on rainstorms and urban flooding are based on geographic information system (GIS) assessments [38], scenario simulation [39], and other model construction methods. Although open and big data have been widely used in urban research, few studies have applied them to rainstorms [40]. Machine learning is an effective tool compared with traditional research methods and is increasingly being applied in rainstorm studies. For example, machine-learning methods and AI techniques for the two-dimensional principal component analysis (2DPCA) method have been used to study the dynamic characteristics of spatial and temporal distributions of rainstorm events in the coastal city of Shenzhen, China, to enable early identification of rainstorm risk [41,42]. Machine learning has also been used to construct a model for flood damage assessment of extreme rainfall events from economic and demographic perspectives [43]. Predictions of urban flooding inundation due to short-duration rainstorms have also been made based on random forest and KNN machine-learning algorithms [44]. Therefore, applying machine-learning methods to VGI is effective for rainstorm research.

Through a preliminary review of the priority proposed by SFDRR and the background of rainstorm-related research, some potential research inadequacies could be noticed. First, the studies focused on spatial risk or impact tend to consider static environmental indicators, which may ignore real-time situations during the disaster process, disaster characteristics, and especially the demands of the affected people. Second, the studies focused on real-time information such as social media posts may tend to belittle the role and value of spatial geographic location information. However, it is necessary to combine static environmental factors with the disaster’s real-time geographic information to identify the disaster characteristics such as vulnerable people and their needs. The widespread use of social media among people in their daily lives in recent times provides more opportunities for determining disaster risk reduction from the perspective of public participation. Hence, by using VGI and machine-learning methods, this paper aims to figure out the following two questions:

(1) How is the characteristic of a severe rainstorm from the perspective of the affected people?

(2) Based on the VGI and environmental indicators, where might be potential shelters with evacuation needs and resources?

By exploring the above two questions, we expect to realize the value and goal of this research from two levels. First, to identify the characteristics of the hazard and possibilities in optimization and enhancement of disaster shelter site selection through the case study of Zhengzhou City for severe rainstorm hazard prevention from the perspective of public participation. Second, beyond the case and the rainstorm hazard itself, to provide some policy recommendations for future emergency management and DRR in the context of the Web 2.0 era. This paper will be described in the following order: In Section 2, the study area, data, and research methods are introduced. In Section 3, the results of this study are presented, including textual analysis of the VGI data collected based on Baidu AI and a Latent Dirichlet Allocation (LDA) model, importance analysis of indicators based on random forest, and construction and prediction of security location models based on binary logistic regression, random forest, and Support Vector Classification (SVC). In Section 4, the findings and possible shortcomings of this study are discussed. In Section 5, a summary of this paper and future research directions are presented.

## 2. Materials and Methods

### 2.1. Study Site

**Figure 1:**
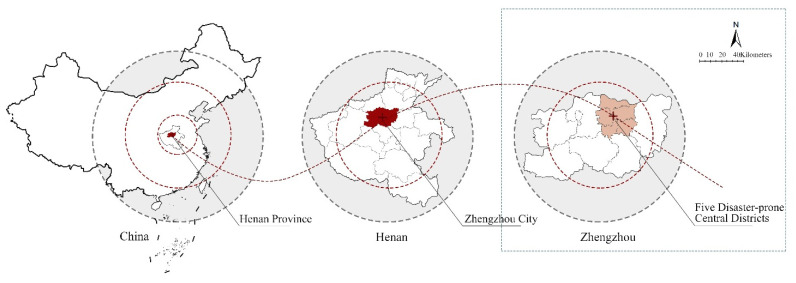
Study site.

Henan Province (110°21′–116°39′ E and 31°23′–36°22′ N) is located in the middle and lower reaches of the Yellow River in east–central China. Zhengzhou City is its capital with six districts (Zhongyuan, Erqi, Guanchenghuizu, Jinshui, Shangjie, and Huiji districts), five county-level cities (Gongyi, Xingyang, Xinmi, Xinzheng, and Dengfeng cities), and one county (Zhongmou County) [45] (Figure 1). Zhengzhou City has a warm temperate continental climate with an average annual rainfall of 640.9 mm.

With an area of 7567 square kilometers and a municipal urban built-up area of 1284.89 square kilometers and an urbanization rate of 78.4%, Zhengzhou is a supercity in central China that has a population of 12.6 million [46]. This kind of high-density city’s disaster response capability and urban resilience level has been the focus of research, especially following the extreme rainstorm disaster on 20 July 2021. Recent studies related to the rainstorm disaster chiefly involve the resilience level evaluation index system of Zhengzhou City [47] and the coupled model of urban built-up area flood forecasting [45]. Research has also focused on the evaluation system of rainstorm safety patterns and land-use strategy under different flood risk levels [48], the joint distribution models of rainstorm elements [49], and urban flood depth prediction [50].

**Figure 2:**
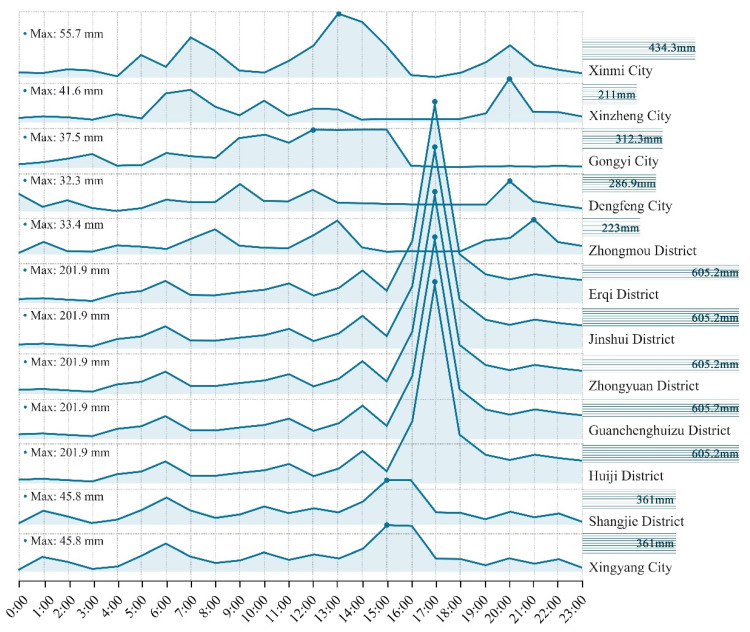
Precipitation from 0:00–23:00 on 20 July 2021, in each administrative region of Zhengzhou City.

On 17–23 July 2021, Henan Province experienced one of the most severe torrential rainfall events in recorded history and consequent severe flooding. Zhengzhou City was particularly severely affected on 20 July. According to information provided by AIRWISE (https://airwise.hjhj-e.com/ (accessed on 16 March 2022), between 0:00 and 23:00 on 20 July, maximum rainfall in Zhengzhou City occurred at 17:00, and cumulative rainfall in Erqi, Jinshui, Zhongyuan, Huiji, and Guanchenghuizu districts (in the central part of the city) exceeded 600 mm (Figure 2). During this disaster, which encompassed 95.5% of the province, 380 people died or disappeared. This severe hazard was defined by the Chinese Government as a rare torrential rainstorm in history. Its intensity and scope broke historical records, far exceeding the urban and rural flood response capabilities. Large areas of urban and rural areas of the city, especially the depressions in urban streets were severely flooded. Many people were trapped in places such as residences, subway stations, or other indoor establishments. Despite the natural factors, the human factors in delay and lack of emergency management were also causes of such major losses that cannot be ignored [51]. In this circumstance, both considering the timeliness and its practical significance for future disaster prevention in cities of similar scale, we chose Zhengzhou City as the study site of this paper.

### 2.2. Collection and Processing of VGI Data and Indicators

The rainstorm began on 17 July and became severe on 20 July, trapping many people on this day. VGI used in this study was obtained from data compiled by volunteers from 20–23 July 2021. Volunteers collected information from social media platforms such as Sina Weibo [52], and WeChat for providing mutual aid during the disaster. They used an online sharing document tool called Shimo (https://shimo.im/ (accessed on 8 February 2022)), sorting the various information into one open-access document accessible to everyone. VGI content was collated for three main areas. First, there were a total of 301 pieces of real-time SOS messages sent by residents during disasters (Table 1).

Second, there were a total of 343 pieces of information on disaster relief that the private sector could provide or temporary water points issued by the government. Third, a total of 241 pieces of information on severely affected areas (such as broken road sections and electricity leakage) were independently reported by citizens. Through manual inspection of the textual information, the addresses in them were screened. Using a tool called “DataMap For Excel”, these addresses were searched on Gaode Map (https://ditu.amap.com/ (accessed on 18 March 2022)) to receive coordinates. These coordinates belonged to the GCJ-02 coordinate system which was not sufficiently accurate with real locations. To ensure the reliability of results, the coordinates of points were converted to the WGS-84 coordinate system for analysis in ArcMap 10.8. Then, each coordinate point was resolved and calibrated from the location obtained from the text as indicator values corresponding to its spatial position. A total of 522 hazardous locations were extracted from VGI using issued SOS messages, including 300 safe locations that were potential evacuation resources for address resolution and subsequent spatial analysis.

In this study, socio-economic [53,54,55], demographic [56,57], and spatial data were also collected. To improve the reproducibility and applicability of the methods proposed, all the data in this research were open data. To improve the data credibility, we also corrected the population data collected by WorldPop based on the results of the seventh census of China. For spatial aspects, topography [58], the number of points of interest (POIs) [50], impervious surfaces [59], and land-use types [53,60,61,62,63,64], were separately selected based on existing studies. We assigned these data to each of the 822 points in VGI to establish a spatial association. We used ArcMap 10.8 to divide the Zhengzhou City area into 250 × 250 m grids [65], and assigned corresponding data separately to each grid. Spatial interpolation was carried out for data with insufficient precision, such as the gross domestic product (GDP), to construct a database with the distribution of hazard and safety locations in the disaster with 25 indicators, as shown in Table 2 [66,67,68].

### 2.3. Methods

**Figure 3:**
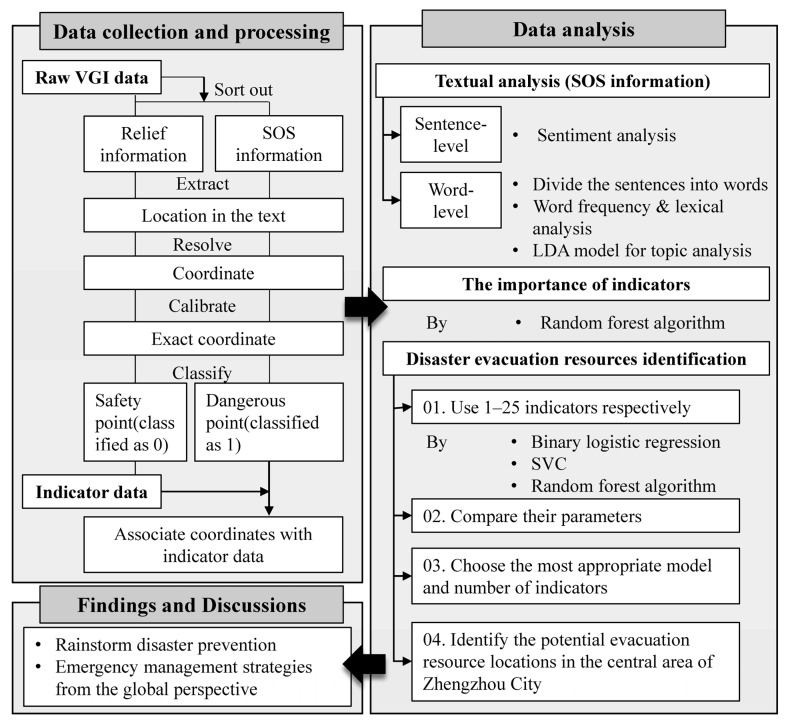
Workflow of the research.

This paper selected the research methods according to the characteristics of the data and the research purpose, whilst considering the methods applied in similar research. For VGI data in the form of text and spatial information, the corresponding methods were employed, respectively. The methods used in this research were determined by the workflow (Figure 3) that reflected the main processes of data collection, processing, and analysis. The first part of the methods was applied to textual analysis for the identification of disaster sentiment and evacuation needs. The second part was applied to measure the importance of influencing indicators and propose suggestions for improving strategies for disaster prevention and disaster risk reduction.

In the first part, the textual analysis was mainly conducted for SOS messages from citizens in the VGI [7]. Three methods were used for identifying evacuation needs. The text of SOS messages from citizens in VGI was divided into words by a Python module named Jieba. Using Python, word frequency and lexical statistics were counted to identify keywords in the SOS messages. The SOS messages were then analyzed for sentiment tendency on a line-by-line basis using Python and the application programming interface (API) of sentiment analysis on the Baidu AI open platform [58] (https://ai.baidu.com/tech/nlp_apply/sentiment_classify (accessed on 12 April 2022). This tool was based on sentiment knowledge enhanced pre-training for sentiment analysis (SKEP), which made it possible to assess the text with a single subject of its subjective information in sentence-level sentiment classification [69]. The output results included the request unique identification code (log_id) of the text, sentiment polarity classification result (0: negative, 1: neutral, 2: positive), probability of belonging to the positive or negative category (value range [0, 1]), and confidence of the judgment result (value range [0, 1]). An LDA model [52,70], was constructed using Python to select the optimal number of topics and content suitable for further understanding of the topic distribution of SOS messages. LDA was a generative probabilistic model and may be used for text corpora. The formula is given below (1) [71]. By establishing a model for the SOS document, the coherence value for each number of topics was calculated and compared. The model with proper coherence value was selected as a suitable model to describe the topic classification for the entire text.

(1) $$p(D|\alpha ,\beta )=\prod_{d=1}^{M}\int p(\theta_{d}|\alpha )\left(\prod_{n=1}^{N_{d}}\sum_{Z_{dn}}p(z_{dn}|\theta_{d})p(w_{dn}|z_{dn},\beta )\right)d\theta_{d}$$

As a three-level hierarchical Bayesian model, there are corpus-level parameters a and b, document-level variables θ*_d_*, and the word-level variables *w_dn_* and *z_dn_*.

In the second part, classifications corresponding to 822 sets of address information, and their corresponding 25 indicators, were used to construct the importance ranking for indicators based on the random forest algorithm [72] using Python. It is a method called random forest variable importance measures(VIMs) that often used to rank candidate predictors [73]. During the initial stages of dataset building, whether a point was safe or dangerous, and the values of the 25 variables associated with that point were known. However, there were differences in the predictive power of these 25 variables for this result; therefore, a random forest model was constructed to compare the degree of influence for the value of each variable on the result. Measurement of the importance of variables was conducive to selecting more important variables as prediction indicators for subsequent model construction and data training, thereby improving the prediction ability of the model.

Based on the indicator’s importance ranking, the optimization of disaster prevention strategies was considered from the perspective of the built-up environment. According to the binary classification nature of point data, binary logistic regression [74], random forest, and SVC [75,76] models are the most common methods for learning and prediction. In this study, three models were constructed with 70% and 30% as the training and test sets, respectively. The results were recorded and compared after placing the 1–25 indicators into the models according to their importance ranking. The best-performing algorithm and the number of indicators were selected according to the parameters for the best prediction model. Five districts (Zhongyuan, Erqi, Guanchenghuizu, Jinshui, and Huiji districts) in the central area of Zhengzhou City had higher rainfall, vulnerability, and susceptibility to flooding than other areas in the city [48] on July 20 and these were used as prediction objects to identify potential safe shelter resource points in the event of heavy rainfall disasters.

## 3. Results

### 3.1. Textual Analysis

#### 3.1.1. Keywords and Sentiments in SOS Messages

Among the 301 SOS text messages, a total of 1898 words were classified. The word frequency and nature were counted, and the most frequent word nature was identified as nouns, verbs, numbers, place names, times, and premises. These reflected the characteristics, number, location, and behavior of the affected people. Words such as “trapped,” “rescue,” “elderly,” “food,” “children,” “power outage,” and “lost” were counted more than 20 times, reflecting the real-time situation of people trapped during the disaster (Table 3).

The 301 SOS text messages were judged by the Baidu AI Open Platform. Among them, 36, 256, and 9 were judged to contain positive, negative, and neutral emotions, respectively. A total of 199 data with >80% confidence level were screened out, including 188 negative sentiments, accounting for 94.47%. This showed that the SOS messages consistently referred to negative emotions, which was in line with the general perception, and indicated that this dataset could realistically reflect the emotions of citizens during disasters. However, these messages also contained some neutral or positive sentiments which reflected that those messages were likely to have not only been sent by the person involved in the disaster but also that some people in a safe situation had sent messages to those trapped in the disaster.

#### 3.1.2. Topics of SOS Messages

**Figure 4:**
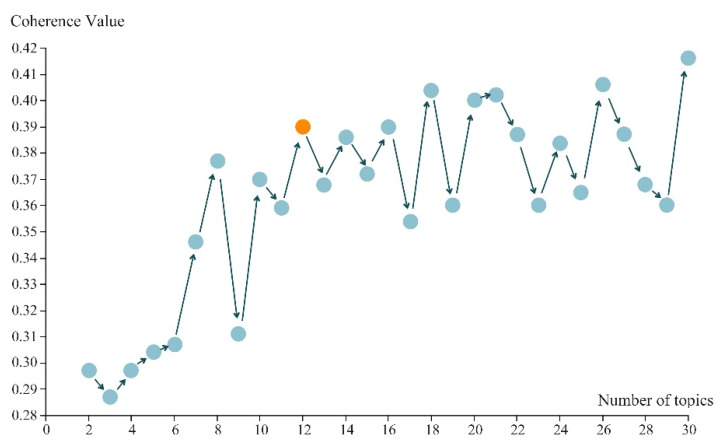
Coherence values of models with a different number of topics (the orange dot indicates the selected number of topics).

Using the LDA method, models with 2–30 topic counts were trained separately and their consistency scores were calculated as a basis for comparison. Consistency scores showed a tendency to fluctuate across the number of topics (Figure 4). A model with 12 topics was selected because it had a relatively high consistency score when N = 12 and was an end of rapid growth in the image. Considering the limited amount of data, the trend of consistency score changes, consistency scores, and the comprehensibility and distinguishability of the output contents of each topic were considered when selecting the model. The number of topics selected did not exceed 15, effectively avoiding the problem whereby more topic count models would have higher consistency scores but also more keyword repetition, which is detrimental to interpreting the topic results.

Table 4 shows the specifics of the model with 12 topic counts. Each topic group was set to be ranked by the 10 most important keywords for that topic and their weighting from highest to lowest. Due to the existence of one unrecognized Chinese character, there were nine keywords in topics No. 2 and No. 7. However, the main content in each topic was not affected. The keywords in each topic reflected the content of that specific topic. SOS message topics involved the needs of vulnerable people such as the elderly [77], children, and pregnant women; shortages of medical resources, water, and energy; terrain characteristics of the location in which people were trapped; and location information. There were also contents such as time spent trapped, the difficulty of rescue, the health status of trapped people, and disaster communication.

### 3.2. Importance of Ranking Indicators and Selection of Forecasting Models

Given that there were 300 safety points with evacuation resources (classified as 0) and 522 dangerous points (classified as 1) in the VGI, the ratio of these two kinds of points was not strictly 1:1. Therefore, the lower limit of the prediction accuracy for this imbalance dataset was calculated as 0.64077; thus, the accuracy of the subsequent model prediction for this dataset was not <64.08%.

**Figure 5:**
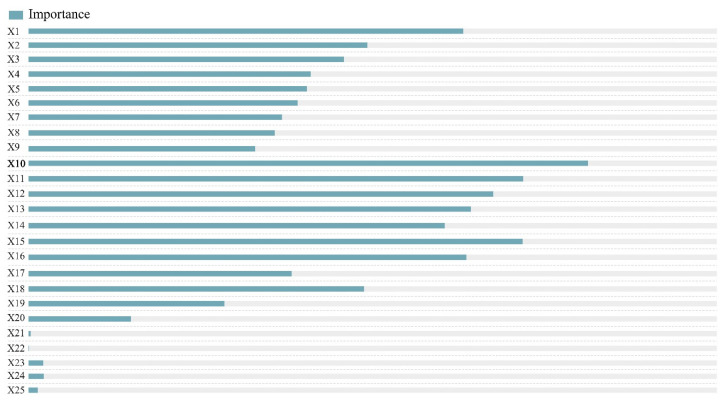
Importance ranking of each indicator based on the random forest algorithm (see Table 2 for the description of X1–X25).

**Figure 6:**
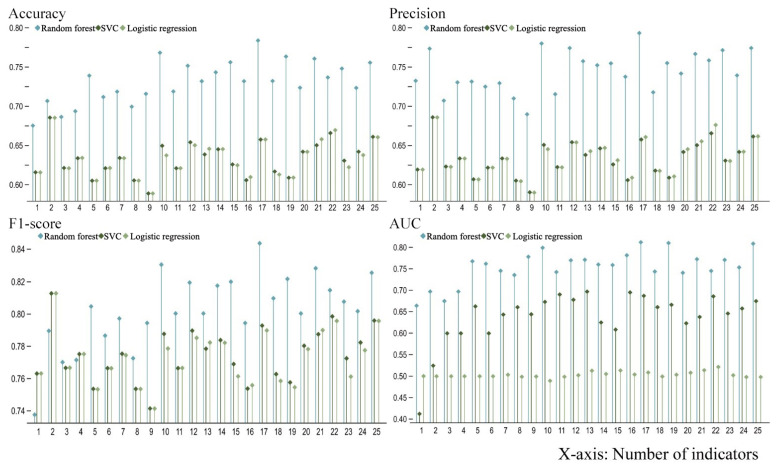
Comparison of the parameters of random forest, Support Vector Classification (SVC), and logistic regression models.

Of the 822 points extracted from the VGI and their corresponding 25 indicator data, 70% were used as the training set and 30% were used as the test set to develop a random forest model. The importance ranking of each indicator was then calculated (Figure 5). Results showed that the planar curvature, elevation, slope direction, slope, and profile curvature in the topography category; GDP and population distribution in the socio-economic and demographic categories; road density and proportion of impervious surface area in the land-use category; and the number of POIs in the grid for living services in the facilities category, had a greater influence on the determination of a point as “dangerous and in need of rescue” or “safe and with evacuation resources.” Logistic regression, random forest, and SVC models were then constructed for this dataset. Given that the SVC model was used, the data needed to be standardized before training was undertaken. The parameters for each model, including the accuracy, precision, recall, F1-score, and the area under curve (AUC) values, were calculated for each of the metrics entered into 1–25 metrics according to their importance ranking. The three models were then compared from the key parameters (Figure 6).

Figure 6 showed that, in terms of accuracy and precision, the random forest model was considerably superior to the logistic regression and SVC models. Although the latter have considerably higher recall than random forest, the recall indicates the probability of a positive sample being predicted in a sample that is actually positive. Therefore, it can only represent the probability when a dangerous point in the original data is judged to be dangerous. A high recall rate indicated that there may be cases in which safe points are also judged as dangerous in real situations. This is a possible misjudgment that is not conducive to distinguishing safety from danger, and a high recall rate may be accompanied by a low accuracy rate. The F1-score indicated that precision and recall were both considered, and the higher the F1-score, the better the model performance. The AUC value can also be used to evaluate the model performance, and the higher the AUC, the better the model. Therefore, among the three commonly used models, the random forest model was the most suitable for this study.

**Figure 7:**
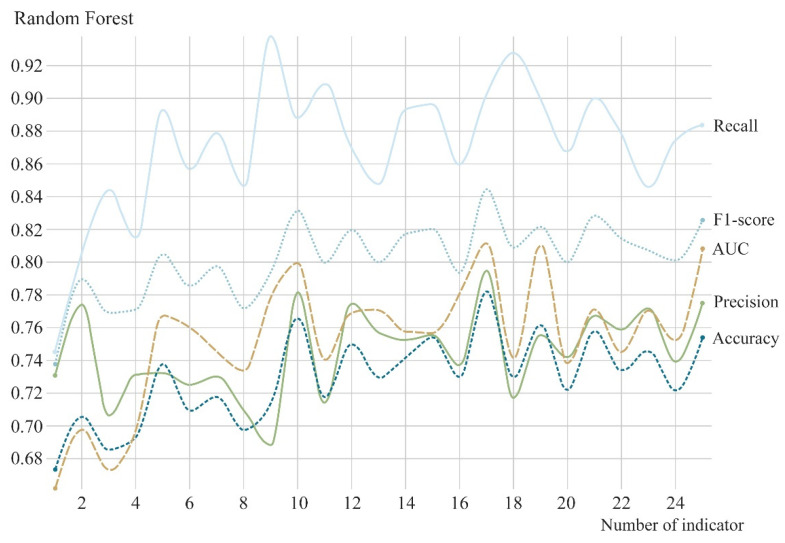
Parameters of the random forest models with different numbers of indicators.

**Figure 8:**
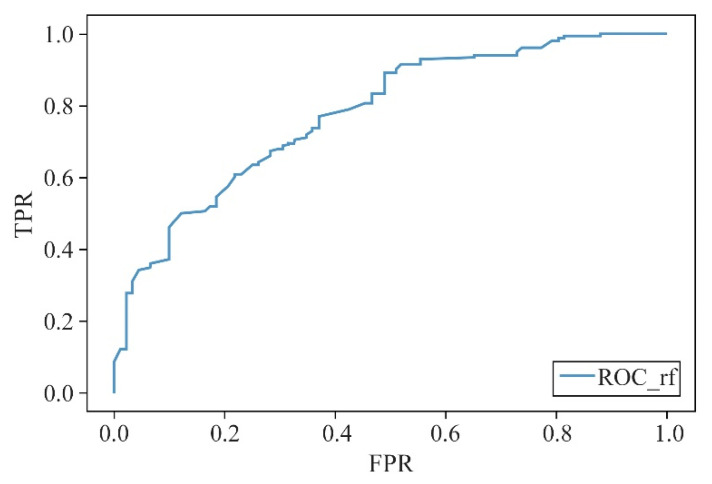
ROC curve for the random forest model with 17 indicators (“TPR”: true positive rate; “FPR”: false positive rate).

After justifying the selection of the random forest model, models with a different number of indicators were compared. Results showed that the model parameters improved after entering the top 17 indicators in terms of importance while having better accuracy (0.7823), precision (0.7945), recall (0.9018), F1-score (0.8448), and AUC (0.8115) values (Figure 7); the receiver operating characteristic (ROC) curve is shown in Figure 8. Therefore, it was determined that the random forest algorithm, when selecting the top 17 ranked indicators in terms of importance, should be used to construct a prediction model for whether the grid within the five districts of the central region of Zhengzhou offered potential safety for evacuation resources.

### 3.3. Optimization of Disaster Prevention through Identifying More Potential Evacuation Resource Locations

**Figure 9:**
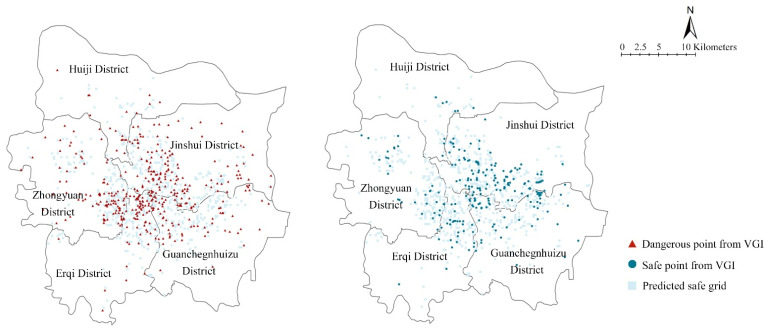
Comparison of predicted safe grids and the dangerous/safe points from volunteered geographic information(VGI).

Using the selected model, grid data for five districts in the central location of Zhengzhou, (Zhongyuan, Erqi, Guanchenghuizu, Jinshui, and Huiji) were analyzed, and 683 potential safety grids were screened. Figure 9 shows the spatial distribution of the predicted safe grids and dangerous points in VGI. Dangerous points rarely coincided with the predicted safe grids, and there was no pronounced spatial correlation. However, the predicted safe grids and safe points in VGI showed certain spatial clustering and correlation characteristics. Therefore, in addition to the scientific nature of the model parameters, it also reflected the credibility of the prediction model constructed from the perspective of visualization.

Compared with the 289 actual security points extracted from the VGI, there were increases in the number and scope (Table 5). The ratio of predicted safe grids to safe points showed that the number of safe grids in each zone was at least 1.45 times higher than the number of safe points in the VGI. There were differences in the ratios between districts. It was predicted that the safe grids in Huiji and Erqi districts increased the most, i.e., by three and four times the number of safe points in the VGI information, respectively. This suggested that there may be more potential shelters and resources in these areas. This result provided a reference for determining safety areas in the region resources could be sheltered during extreme storm disasters. This compensated for the problem of having insufficient real-time VGI of an area during the disaster and, consequently, being unable to judge the safety of that area. For existing dangerous points, the decision-makers need to figure out why people here got trapped, then strengthen their disaster response capacity. For predicted safety points, along with the verification of their actual situation, some new potential shelters can be found. The urban emergency system can be optimized based on the current emergency shelters.

## 4. Discussion

Through the case study of the severe rainstorm that occurred in Zhengzhou City, it is shown that VGI has a certain level of reliability as a data source. The VGI issued by residents during the disaster reflected realistic content, themes, and emotions of the people in distress involved in the rainstorm in Zhengzhou. The number of SOS messages was much higher in the central areas of Zhengzhou than in the suburbs, which is consistent with previous studies that found higher flood risk in the central and old urban areas of Zhengzhou [48]. In the meanwhile, based on the indicators selected from similar disaster risk studies, this study focused on five aspects (topography, socio-economics, population distribution, public facilities, and land use) to investigate the extent to which a geographic location with VGI was judged to be “safe and has evacuation resources.” In the case of Zhengzhou, the predicted 683 security grids were more than the 289 original security points obtained based on VGI for the five districts of the central region. Therefore, the actual security area was considered to be larger than that reflected by the VGI. In the context of future disaster prevention and mitigation work, the optimal layout of evacuation sites can be achieved according to these 683 grid points. The areas outside the safety points can also be a focus for hidden danger investigation and the optimization and enhancement of disaster prevention capabilities.

In addition to the case itself, there is actually more content worthy of discussion and attention in further rainstorm studies in the future. First, it is the number and quality of VGI. VGI data are free, open, and timely, and can provide first-hand information for disaster studies [78]. However, the accuracy and information distribution of such data may not be ideal due to factors such as personnel distribution and limitations associated with communication. Consequently, the performance of the trained model may not be as effective as the results based on professionally collected data. Furthermore, the sample size for disaster studies using LDA models is generally large [79,80]. In future studies, the obtained results could be better by collecting a larger number of samples for textual analysis.

Second, the difference between urban and rural areas should not be ignored, especially in developing countries. On the one hand, due to the difference in population distribution and economic development levels between the urban and rural areas, it was found that VGI data were more easily gathered from urban areas, which provided enhanced the possibility of getting a more accurate result for the urban areas than the rural areas. Since the urban areas have a higher population density and built area, their vulnerability is consequently higher. However, disaster damage in undeveloped rural areas should not be neglected. Therefore, the manner in which this research framework can be applied to undeveloped areas should be considered in future research. On the other hand, when selecting the important predictors, the POI category accounted for a larger proportion of the top-ranked indicators. This may have led to the model having a more accurate prediction capacity for urban areas with more complete POI information, especially the central city. Although POI can effectively reflect the built-up environment (especially land use) for areas with incomplete POI data (e.g., rural areas), it is possible that the model might not perform effectively in rural areas. In future studies, the model can be further optimized and improved in terms of indicator selection.

Third, the value of VGI generated during hazards should gain more attention from the authorities. Compared to the traditional big data applied to emergency management, the usage of VGI should also be viewed as a promising data source in the context of Web 2.0 and the wide use of smartphones. The decision-maker such as the government should take measures including cultivating and training specialized volunteers, building a real-time public disaster information-sharing platform, and formulating corresponding emergency plans, etc. Policies should also be developed to ensure that VGI data contribute to disaster prevention to the greatest extent possible.

## 5. Conclusions

The Web 2.0 era provides more opportunities and possibilities for the optimization of big data in disaster prevention and emergency management. In the context of priorities proposed in the SFDRR, this study preliminarily reviewed the current state of research on extreme rainstorm hazards, and the related technical background. Through the case study of a severe rainstorm hazard in Zhengzhou City, China, the possibility of applying VGI and machine learning in extreme rainstorm hazard research was explored. The policy recommendations on DRR and strategies for future rainstorm hazards research and disaster prevention were also discussed. The main conclusions are summarized as follows.

First, VGI should receive more attention during disaster research in the future. Mutual aid information during disasters collated by volunteers as VGI can serve as a reliable data source [81]. It provides a descriptive account of the disaster in real time, further validating the idea that VGI data can help reproduce the real-time dynamics of disasters [82]. VGI data analysis and application can help compensate for an inadequate understanding of the actual situation during these disasters. This study showed that SOS information, even from non-professionals, analyzed by information extraction, LDA modeling, and other textual analyses could provide valid information, including emotional tendencies, needs, and locations, and can be used as a reliable data source for further spatial analysis.

Second, vulnerable people and their demands should gain more attention from the authorities. The disaster-vulnerable populations in extreme urban rainstorm disasters were predominantly the elderly, children, and pregnant women. Most of the demands were associated with loss of water and electricity, lack of food and drinking water, being trapped in transportation, poor communication, and instability of buildings. Such claims should be treated as priorities in preparation for future disaster responses for the expeditious protection of vulnerable people and to strengthen infrastructure construction and necessary material stockpiles to reduce human casualties and property damage in the event of a natural disaster.

Third, it is necessary to combine VGI with other authoritative or open-source data in emergency management to reduce emergency response times and improve disaster resilience in cities. In terms of rainstorm hazards, the topography, population distribution, economic development level, and the built-up environment of a city exhibited different degrees of correlation with the impacts caused by extreme rainstorm hazards. Especially the section and plane curvature, elevation, slope and slope direction of typographic elements, the average land GDP, population of socio-economic elements, the POI distribution, road network density, and the percentage of impervious surfaces in the built-up environment all had relatively important effects on the classification of hazard and safety points in rainstorm disasters. In contrast, indicators regarding land-use types (e.g., water, trees, or grass) may be relatively unimportant indicators, which provides a reference for indicator selection in future urban rainstorm hazard studies.

Last, to make full and efficient use of VGI information generated by ordinary people during disasters, thereby reducing disaster risks and losses caused by disasters, there are still many areas worthy of optimization in terms of policies. As a researcher, it is necessary to further understand public participation in disasters, especially the public’s preferences and habits for risk communication and social media use during disasters, to make a more accurate analysis. Besides, decision-makers should pay attention to the timely information generated by the public during disasters, and build a more convenient platform for the release of such information. In addition, the processing, presentation, and dissemination of data should be strengthened, to effectively reduce disaster risks and improve the comprehensive ability of emergency management.

## Figures and Tables

**Table 1:** Examples of SOS messages.

Example 1	Example 2
Is there anyone at Zhengzhou East Station? Friends are trapped inside the station! The little girl has a fever and a severe headache. She does not know what to do now without medicine. There is not much battery left on her mobile phone. Contact number: XXX-XXXX-XXXX.	Address: 2 km north of Sanglin Road, Zhengkai Avenue, Zhengzhou City, in Hengze Logistics Park. Hundreds of people have been trapped for 24 h, the water level is still rising, people are already on the roof, and there is no way out! No water, no power, no food. Some people already feel unwell and ask for rescue, emergency! Urgent!

**Table 2:** Indicators and data sources of spatial factors.

Category	Indicator	Description	Data Source
Distribution of facilities	(X1) POI of domestic services	Number of living service facilities in the grid	Gaode Open Platform (https://lbs.amap.com/ (accessed on 18 March 2022))
	(X2) POI of dining and shopping	Number of dining and shopping facilities in the grid	
	(X3) POI of transportation facilities	Number of transportation facilities in the grid	
	(X4) POI of sports and leisure facilities	Number of sports and leisure facilities in the grid	
	(X5) POI of government organizations	Number of government agencies in the grid	
	(X6) POI of science and education facilities	Number of science, education, and cultural facilities in the grid	
	(X7) POI of industry and enterprises	Number of industrial and business facilities in the grid	
	(X8) POI of financial institutions	Number of financial facilities in the grid	
	(X9) POI of medical institutions	Number of medical facilities in the grid	
Typography	(X10) Section curvature	Section curvature at grid center point	Calculated using ArcGIS 10.8 on 30 m DEM data from Geospatial Data Cloud.
	(X11) Elevation	Elevation at the grid center point	
	(X12) Plane curvature	Plane curvature at grid center point	
	(X13) Slope direction	Slope direction at grid center point	
	(X14) Slope	Slope at grid center point	
Society and economy	(X15) GDP	GDP of the grid (in 2015) 1 km resolution data with spatial interpolation	Kilometer grid dataset of China’s GDP spatial distribution. The data were obtained from the Resource and Environmental Science Data Registration and Publishing System [66]
	(X16) Population	The population of the grid Population counts/constrained individual countries 2020 UN adjusted (100 m resolution) Population data were corrected according to Official data from China’s seventh population census	WorldPop [67]
Land use	(X17) Proportion of impervious surface area	Percentage of impervious surface in the grid	GISD30: global 30 m impervious surface dynamic dataset from 1985–2020 [68]
	(X18) Road density	The density of roads in the grid	Baidu Map Open Platform
	(X19) Water	Area of water in the grid	ESRI: Sentinel-2 10-Meter Land Use/Land Cover
	(X20) Built area	Area of built area in the grid	
	(X21) Bare ground	Area of bare ground in the grid	
	(X22) Trees	Area of trees in the grid	
	(X23) Crops	Area of crops in the grid	
	(X24) Grass	Area of grass in the grid	
	(X25) Shrub	Area of shrubs in the grid	

POI—point of interest; DEM—digital elevation model.

**Table 3:** Keywords counted more than 20 times in SOS messages.

No.	Word	Count	Flag	No.	Word	Count	Flag
1	trapped	172	adjective	15	water lever	32	noun
2	rescue	103	verb noun	16	rescue team	31	noun
3	hour	65	noun	17	Zhengzhou	29	place
4	the aged	63	noun	18	20	29	numeral
5	child	62	noun	19	hope	29	verb
6	food	47	noun	20	personnel	27	noun
7	cell phone	45	noun	21	signal	26	noun
8	telephone	45	noun	22	water cut off	24	verb
9	not accessible	39	adverb	23	condition	23	noun
10	community	37	noun	24	friend	23	noun
11	power failure	36	verb	25	on the car	22	place
12	help	34	verb	26	no power	22	verb
13	urgent need	33	noun	27	materials	21	noun
14	lost contact	32	verb noun	28	stagnant water	21	noun

**Table 4:** Topic model with 12 selected topics.

Topic No.	Keywords and Their Weights	Topic Summary
1	0.033 × “the old” + 0.032 × “urgent need” + 0.021 × “hour” + 0.019 × “generator” + 0.012 × “one” + 0.011 × “friend” + 0.009 × “mobile” + 0.009 × “company” + 0.009 × “worry” + 0.008 × “information”	Vulnerable people, needs
2	0.213 × “call for help” + 0.206 × “scenic area” + 0.128 × “trapped” + 0.109 × “!!” + 0.003 × “reservoir” + 0.003 × “area” + 0.003 × “place” + 0.003 × “support” + 0.003 × “section”	Trapped location
3	0.034 × “!” + 0.017 × “child” + 0.015 × “Zhengzhou” + 0.015 × “power outage” + 0.012 × “flooded” + 0.011 × “water outage” + 0.011 × “first floor” + 0.011 × “month” + 0.011 × “day” + 0.010 × “food”	People, time, location, needs
4	0.021 × “inside” + 0.017 × “transfer” + 0.016 × “condition” + 0.016 × “water cut off” + 0.013 × “hope” + 0.012 × “dad” + 0.011 × “one person” + 0.008 × “battery” + 0.008 × “tumor hospital” + 0.007 × “medical staff”	Medical resources
5	0.017 × “year old” + 0.017 ×“the old” + 0.013 × “water level” + 0.012 × “less than” + 0.012 × “home” + 0.011 × “water depth” + 0.011 × “landslide” + 0.011 × “occurrence” + 0.010 × “one meter” + 0.009 × “baby”	Vulnerable people, secondary disaster
6	0.012 × “message” + 0.011 × “phone” + 0.010 × “terrain” + 0.010 × “front” + 0.010 × “five o’clock” + 0.010 × “Zhengzhou” + 0.010 × “multiple people” + 0.010 × “please“ + 0.010 × “point” + 0.009 × “height”	Information, topography, population characteristics
7	0.018 × “hotel” + 0.018 × “power outage” + 0.014 × “water outage” + 0.014 × “thank you” + 0.014 × “signal” + 0.013 × “transfer” + 0.012 × “water” + 0.012 × “children” + 0.010 × “the old”	Location, energy, transfer
8	0.058 × “rescue” + 0.034 × “trapped” + 0.023 × “community” + 0.020 × “no” + 0.017 × “person” + 0.017 × “water” + 0.015 × “old people” + 0.014 × “20” + 0.012 × “request” + 0.012 × “night”	Location, needs, time
9	0.015 × “urgent” + 0.015 × “road” + 0.014 × “no access” + 0.014 × “yesterday” + 0.013 × “home” + 0.011 × “family” +0.011 × “rescue team” + 0.011 × “phone” + 0.011 × “cannot get out” + 0.010 × “place”	Rescue
10	0.145 × “trapped” + 0.116 × “help” + 0.115 × “submerged” + 0.112 × “parking lot” + 0.011 × “hospital” + 0.010 × “tears” + 0.010 × “pregnant women” + 0.009 × “hours” + 0.008 × “floor” + 0.008 × “waiting”	Location, affected people
11	0.061 × “!” + 0.033 × “water” + 0.022 × “car” + 0.020× “food” + 0.015 × “method” + 0.014 × “community” + 0.012 × “thank you” + 0.009 × “help” + 0.008 × “fever” + 0.008 × “true”	Mood, materials, health condition
12	0.021 × “no access” + 0.020 × “no” + 0.020 × “mobile” + 0.016 × “no contact” + 0.015 × “no power” + 0.014 × “help” + 0.013 × “eat” + 0.010 × “hours” + 0.010 × “shutdown” + 0.009 × “bad”	Contacts

**Table 5:** Comparison of predicted results and actual data.

District	Dangerous Points from VGI	Safe Points from VGI	Predicted Safe Grids	Ratio of Predicted Safe Grids to Safe Points
Erqi	76	39	117	3.00
Guanchenghuizu	84	61	139	2.28
Jinshui	156	140	203	1.45
Zhongyuan	110	40	143	3.58
Huiji	27	9	36	4.00
Total	453	289	638	2.21

## Data Availability

All data used in this study are openly available. Some data are available in a publicly accessible repository that issues DOIs, whereas some are available in a publicly accessible repository that does not issue DOIs. The method to access the data in this study can be found in the article at the place where the data are first mentioned.
